# Impact of B cells to the pathophysiology of multiple sclerosis

**DOI:** 10.1186/s12974-019-1517-1

**Published:** 2019-06-25

**Authors:** Borros M. Arneth

**Affiliations:** Institute of Laboratory Medicine and Pathobiochemistry, Molecular Diagnostics, University Hospital of the Universities of Giessen and Marburg UKGM, Justus Liebig University Giessen, Feulgenstr. 12, 35392 Giessen, Germany

**Keywords:** Multiple sclerosis, Experimental autoimmune encephalitis, B cells, B lymphocytes, Plasma cells, Antibodies

## Abstract

**Introduction:**

Multiple sclerosis (MS) is a chronic autoimmune disorder that affects the central nervous system and compromises the health and well-being of millions of people worldwide. B cells have been linked to MS and its progression. This review aimed to determine the role of B cells in MS development.

**Methods:**

Articles used in this review were obtained from PubMed, LILACS, and EBSCO. The search terms and phrases included “multiple sclerosis,” “MS,” “B-Cells,” “pathogenesis,” and “development.” Original research studies and articles on MS and B cells published between 2007 and 2018 were included.

**Results:**

Results from the selected articles showed a significant connection between B cell groups and MS. B cells act as a significant source of plasma cells, which generate antibodies while also regulating autoimmune processes and T cell production. In addition, B cells regulate the release of molecules that affect the proinflammatory actions of other immune cells.

**Discussion:**

B cells play key roles in immune system functioning and MS. The findings of this review illustrate the complex nature of B cell actions, their effects on the autoimmune system, and the method by which they contribute to MS pathogenesis.

**Conclusion:**

Previous research implicates biological, genetic, and environmental factors in MS pathogenesis. This review suggests that B cells contribute to MS development and advancement by influencing and regulating autoimmune processes such as T cell production and APC activity.

## Introduction

Multiple sclerosis (MS) is a chronic autoimmune disorder that affects the central nervous system. In 2015, approximately 2.3 million people had MS globally [1]. The disease onset usually occurs between the ages of 20 and 50 years, and it is twice as common in women as in men. MS was first described in 1868 by Jean-Martin Charcot, and since then, several forms of the disease have been identified [2, 3]. Between different MS stages, patients experience symptoms with varying degrees of severity. In most cases, people with MS face permanent neurological problems that affect their everyday life. MS progression is characterized by different signs, such as white matter plaque formation, axonal injury, and demyelination, which mainly occur in the spinal cord, optic nerve, brain stem, and periventricular regions [4, 5]. The signs and symptoms of MS vary depending on the affected part of the CNS. For example, motor, sensory, visual, and autonomic dysfunction present when the cerebrum, brainstem, visual pathway, spinal cord, and cerebellum are affected [6–8]. Other symptoms of MS relapse are extreme weakness and bowel, cerebellar, and bladder dysfunction with pyramidal tract involvement [9–11]. However, MS relapse that is linked to pyramidal signs, sphincter dysfunction, or cerebellar dysfunction is more severe and must be treated promptly [12–14].

Presently, multiple sclerosis has no known cure. However, caregivers strive to conduct thorough examinations to identify symptoms that can be managed and treated [15, 16]. The most important tool for evaluating MS is a physical examination, which involves assessing significant signs to evaluate changes in the affected individual’s blood pressure, heart rate, and temperature [17–19]. The neurological examination involves assessing strength, vision, coordination, gait, and sensation. In other cases, vision testing includes examining eye movements, visual acuity, visual fields, and color vision. Treatments attempt to improve function after an attack and prevent new episodes [20–23]. Medications are also used to manage MS despite their side effects that may adversely affect the patient [24]. In other cases, caregivers use physical therapy to improve functioning among those with MS [25]. These interventions aim to relieve MS symptoms, slow disorder progression, and save individuals from developing further disability.

Studying the development of different immunological conditions such as MS can be complex and challenging. The exact cause of MS development is unknown [26]; however, an amalgamation of infectious agents, environmental concepts, and genetics is believed to be the main causes [27–29]. Over the years, genome-wide investigations have implicated several gene variants in MS development. Most of these genetic variants encode a wide range of molecules that participate in immune responses [30, 31]. The results of such studies have supported the notion that MS is an immunologically mediated disorder. More recent studies have examined the way different environmental risk issues and factors contribute to MS emergence [32–35]. The topics and causes that have been studied include viral infections, vitamin D levels, smoking, and obesity. Interactions between environmental and genetic factors are implicated in MS emergence in patients [36, 37]. A large amount of research and evidence implicates different bodily molecules and components, such as B cells, in MS pathogenesis [37]. B cells play key roles in the normal immune processes and bodily responses [38]. The effects of B cells on antibody production and the workings of the adaptive and innate immunological responses have been linked to MS. This paper aimed to explore the contributions of B cells in MS.

### Methodology

This review analyzed evidence that describes how B cells affect MS development. Articles that examined the association between B cells and MS were identified. The articles were obtained from electronic databases, including PubMed, LILACS, and EBSCO. All databases were searched using an identical strategy and search terms. In this case, the search terms included “Multiple Sclerosis,” “MS,” “B-Cells,” “pathogenesis,” and “development.” Boolean operators were used in the search process to combine the terms and locate additional articles. The search was limited to original research studies and articles on MS and B cells that were conducted in humans and published in English from 2007 to 2018. Additional articles were obtained by reviewing the bibliographies of the already identified reports. The abstracts of the identified sources were carefully examined to assess their relevance to the present study. Records that met the inclusion criteria were reviewed in full, and the credibility of each study’s authors, objectives, methodologies, results, discussions, conclusions, and limitations was determined. At the end of the search process, many studies using different methodologies were included in the final list. Data were collected by summarizing the articles and comparing the findings on the association between B cells and MS.

## Results

### Types of B cells

**Fig. 1:**
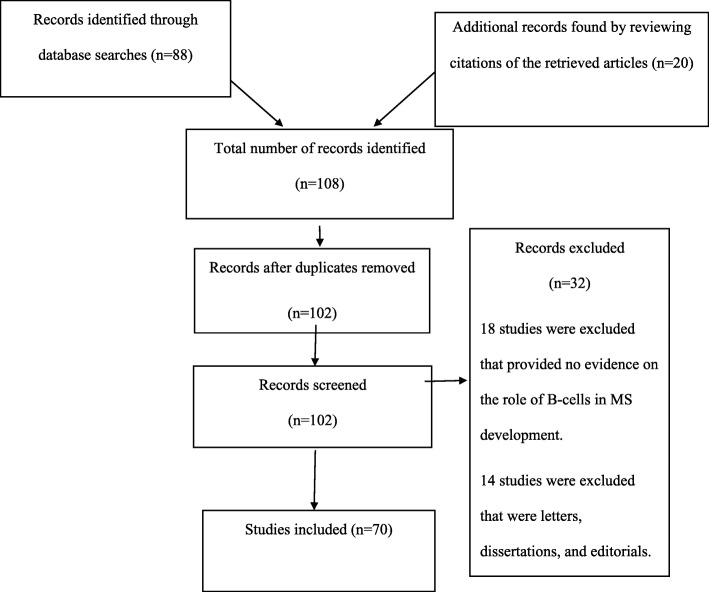
Prisma flow diagram

Previous studies have identified different B cell types. The first are plasmablasts, which are largely antibody-secreting cells formed through differentiation [30]. Plasmablasts are usually formed in the early stages of an infection and have a lower affinity towards the target antigen. In some cases, the cells are formed via extrafollicular activation. Second are the plasma cells, which differentiate into plasmablast-like cells. They can be formed in the later stages of the infection and have a higher affinity to the target antibody. The third group are the lymphoplasmacytoid cells, which are a mixture of plasma cells and blasts [31]. Fourth are memory B cells, which usually arise from B cell differentiation and promote rapid antibody response. Other B cell types identified in previous studies include B1 cells and regulatory B cells. The literature search was done following the PRISMA flow diagram given in Fig. 1.

### B cells and MS

MS causes emotional, physical, health, and economic burdens to patients, their families, societies, and nations. This study examined the role of B cells in MS [38]. Evidence collected from previous studies showed that MS emergence is influenced by extensive factors such as gene variants, vitamin D levels, lifestyle, and infectious diseases. Studies have revealed that most causative factors are modifiable and necessitate attention from both patients and caregivers to achieve favorable outcomes [39]. Although the actual mechanism underlying the link between the identified factors and MS is unclear, caregivers should understand the MS development process. One factor that healthcare practitioners should focus on is the role played by B cells in advancing the condition.

Recent studies have resulted in the emergence of a new conceptual framework for MS development and pathogenesis [40]. This new approach and understanding focus on the function of anti-CD20 antibodies in influencing MS cases [39, 40]. These results have increased researchers’ attention on the possible effects of B cells in autoimmune disorders such as MS [40]. Autoreactive B cells exist in the immunological systems of healthy persons [41]. These cells have critical physiological functions in normal autoimmunity. Deficiencies in these cells can affect the immunosuppressive functions in the body and result in the emergence of abnormalities such as MS and rheumatoid arthritis.

### B cells target autoantigens

Research indicates that B cells affect MS development and progression by targeting autoantigens [42–45]. In addition, humoral antibodies are reported to lead to tissue injury when they bind to brain cells and interfere with complement factor functions. More recently, leptomeningeal B cells were found to cause neuronal degeneration and demyelination [32]. In addition, B cells can deplete anti-CD20 antibodies, causing MS relapse and further neurological deficiencies. However, the target antigens in MS development remain an issue of debate and research. Despite this, B cells contribute significantly to MS development and progression.

Studies have revealed that oligoclonal immunoglobulin (Ig) persists in the cerebrospinal fluid (CSF) in approximately 90% of patients, further supporting the idea that B cells contribute to MS pathogenesis [46]. Ig that is intrathecally produced by plasma cells is a hallmark in diagnosing and managing MS. Recent comparisons of transcriptomes of CSF B cells and CSF Ig proteomes revealed that clonally expanded B cells in the CSF usually produce oligoclonal bands (OCBs). Further molecular analysis of B cells has shown that maturation of their antigen-driven affinity in the CSF can lead to somatic hypermutation [33]. Despite intensive investigations, researchers have yet to reach a conclusion on the manner in which antibodies recognize antigens during MS development and progression. However, the humoral immune response process involves the production of antibodies that fight neurotropic viruses, indicating that no particular antigen facilitates OCB development in patients with MS. Moreover, evidence shows that no particular mechanism activates CSF-localized B cells among patients with MS [40].

Evidence from histological studies shows that Ig colocalization and deposition in areas of CNS demyelination are central to MS development. In addition, CSF-based antibodies usually cause axonal damage while also facilitating the complement-mediated demyelination process. These antibody responses may target antigens such as myelin oligodendrocyte glycoprotein (MOG), myelin basic protein, neurofascin, and contactin-2 during MS emergence [34]. Furthermore, humoral responses damage the CNS via the action of intracellular epitopes such as on DNA and RNA. The pathogenic action of the CNS-based antibodies is usually characterized by enhanced inflammatory demyelination and blood-brain barrier disruption. CNS-directed antibodies were recently reported to affect pathogenic functions outside the CNS [35]. In some cases, studies using animal models have indicated that peripheral antimyelin antibodies can activate myelin-reactive T cells. This sequence of responses can also be triggered by the opsonization of CNS antigens in the body.

### Evidence from animal models on the influence of B cells in MS

Antigen-activated B cells in the body can facilitate MS development by acting as potent antigen-presenting cells (APCs). Furthermore, B cells usually act as a source of antibody-generating plasma cells to contribute to MS development and progression [36]. This argument has been supported by studies revealing that anti-CD20-mediated B cell depletion is central in MS development. Peripheral CNS B cells usually contribute to chronic inflammation [37, 47]. B cells found in MS patients are usually characterized by the expression of costimulatory molecules, an event contributing to the emergence of inflammatory demyelinating disorders such as MS and experimental autoimmune encephalomyelitis (EAE).

Recent animal studies showed that B cells usually act as a source of both pro- and anti-inflammatory cytokines [48, 49]. In addition, naïve and activated B cells are considered potent producers of protective and pathogenic cytokines. B cells are involved in regulating other immune cells that affect inflammatory responses. Investigations show that B cells can produce IL-6 and aid the process of T helper-17 cell differentiation. Furthermore, they prevent the production of regulatory T cells [38]. Animal model studies have evidenced that B cells indicate IL-6 deficiency, which can reduce MS severity [38, 39]. Peripheral B cells can increase the secretion of many inflammatory factors such as IL-6, tumor necrosis factor (TNF), and lymphotoxin-α (LT-α). More interestingly, the cells facilitate proinflammatory B cell responses such as polyclonal stimulation in MS patients. The other proinflammatory molecule that B cells produce during MS development is the granulocyte-macrophage colony-stimulating factor (GM-CSF) [39].

Human and animal studies show that GM-CSF-generating B cells can also facilitate IL-6 and TNF expression. Furthermore, deleting these cells usually decreases myeloid cell pathogenic immune responses.

B cells also contribute to MS development by producing many anti-inflammatory cytokines. Some molecules linked to this process include transforming growth factor-β1, IL-35, and IL-10. Furthermore, these cells can generate large amounts of IL-10, a process that compromises the actions of various myeloid APCs. In some instances, IL-10 generation affects the functioning of dendritic cells and inhibits the process of TH1 and Th17 differentiation [40]. Recent experimental investigations have shown that the cytokines produced by B cells are central to preventing autoimmune attacks that affect CNS functions [41, 42]. Furthermore, mice deficient in B cell-linked IL-35 and IL-10 may not recover from autoimmune attacks. In addition, the increase in IL-17 and interferon-γ (IFN-γ) production may result in increased MS severity [41–43]. Results have been linked to the critical role of B cells in regulating immunological synapses and T cell production. This mechanism is further supported by blood samples obtained from MS patients, which contained B cells that could cause anti-inflammatory actions and regulate monocytic activity.

B cell homeostasis and function in the central immune system are pertinent to comprehending MS pathogenesis [38]. Research shows that MS patients often have augmented proportions of peripheral B cells and VLA-4 receptors [42, 43]. These are critical molecules that affect MS progression and influence its severity. Increased cell attractant chemokine, CXCL13, VH2, and VH4 have further been reported in MS patients [44, 45]. Existence of these molecules indicates that a broad spectrum of B cell populations can influence MS progression. In other cases, researchers suggest that B cell biomarkers and activation correlate with MS advancement in some people [44, 45]. For example, CXCL13 has been linked with progressive MS. In other cases, research has revealed that CXCL13 determines the degree of MS and its activity among patients [46]. Documentation of inflammatory variants linked with B cell germinal points has supported a possible connection between B cell populations and MS [48].

B cell subpopulations are critical to improving the well-being of MS patients. In addition, B cells affect functional recovery and the spread of inflammation in MS patients. The process usually involves immune system activity. However, a key question that remains is the manner in which B cell functions may be exploited and targeted to enhance patients’ well-being. Evidence from early studies associates the production of molecules, such as IL-10, to the naïve B cell population [43, 44]. Recent animal models indicated that antigen-experienced B cells may also affect plasma cell differentiation and the generation of IL-10, IL-35, and regulatory B cell cytokines [46, 47]. These molecules have important anti-inflammatory properties that may affect MS progression.

### B1B cells in MS

B1B cells can act as surface immunoglobulin receptors. Under favorable conditions, these cells can differentiate into plasma cells and produce antibodies that can assist in preventing infections and regulating MS progression [48, 49]. In addition, B1B cells have additional activities that facilitate producing secondary signals during MS infection. Therefore, B1B cells are central in modulating immunological responses during MS development and progression. B1B cells are a subset of B cells that limit the chances of relapse among MS patients [50]. The existence of the B1 cells in the body has been inversely correlated with disease progression [48]. More recently, researchers have stated that the B1B cells can spontaneously produce IgM antibodies and interact with the prime T cells [49, 50]. In addition, these cells can affect disease progression by influencing CD11b production and expression [41, 51–53]. Recent studies have shown that these cells may also cause preplasmablast differentiation to influence MS progression.

The impact of B1B cells on MS development has further been examined in studies focusing on the subsets that can produce cytokines and exert anti- or proinflammatory actions. B cells are vital source points of CNS antibodies and plasma cells [54–56]. Moreover, they can regulate and control inflammatory actions through different cytokines. In some cases, B1B cell populations facilitate generating Th17 cells by increasing IL-6 levels in the body [57]. However, not all B cells lead to disorders affecting the immune system. On the contrary, these cells influence a wide range of inflammatory processes that can either hinder or encourage the advancement of these disorders. These functional dichotomies have been established in studies focusing on B cell groups such as peritoneal B1 or follicular B2 cells [32, 37]. These cell categories differ from conventional ones in terms of antibody influence, location, and genetic expression [37]. The B1 subsets are primarily domiciled within the peritoneal cavity and participate in autoimmune functions. Their actions are influenced by many processes including the expression of potent antigens. The category of B1 determines the generation of different molecules, such as Th1 cells, which affect MS growth [55]. In contrast, the B2 cells aid the generation of regulatory T cells, which are known for their unique suppressive capabilities [58, 59].

The occurrence of uncharacteristic rates of immunoglobulin synthesis in the body is regarded as a hallmark and indicator of MS. Scholars report that immunoglobulin G (IgG), HLA-G, and CD200/CD200R can be found in patients with MS [49–51]. These molecules exist in approximately 30–40% of persons with MS and are linked to active diseases. Furthermore, these substances indicate the possible role of B cells in MS advancement [52, 53]. The findings from this study suggest that B cells and immunoglobulin production can influence MS and affect the patient’s response to therapy and treatment [54]. Cells isolated from samples from MS patients have further been found to produce specific antibodies [55, 56]. Recent studies based on somatic hypermutation experiments showed that MS patients experience bidirectional movement of B cell population clones that affect disease symptoms and advancement [55]. Interestingly, studies postulate that subsets of B cells may affect MS relapse and progress through drainages into the lymph nodes and affect peripheral lymphoid tissues [56]. Despite this, little is known about the actual mechanism through which B cell-linked molecules and antibodies affect the disease.

Research indicates that B cells found in humans can influence CNS functioning through their protective functions and pathogenic effects. Traditionally, MS has been viewed as a disease that is largely influenced by the actions of T cells [57]. However, recent research shows that the condition is antibody-dependent and propagated by B cell functions. By acting on the CNS and the peripheral disorder compartments, B cells determine the symptoms that MS patients experience. Recent in-depth examination of the immunoglobin makeup in MS patients resulted in identifying various intracellular self-proteins that suggest dead cell debris presence and injury [58, 59]. Scholars contend that determining the pathogenic roles of B cell-linked antibodies in MS development is challenging [60]. Evidence from early studies used detection tools such as immunosorbent assays to demonstrate the influence of molecules, such as myelin antigens, in MS emergence [61]. Even with these tools, identifying the specific molecules that affect particular processes in MS remains difficult and complex.

Some MS researchers have examined B cell functions by focusing on their function as T cell activators. B cells influence immune responses when they differentiate into numerous antibody-producing plasma cells [61]. However, they can also affect MS progression by stimulating T cells. Evidence from previous studies has implicated T helper (Th) 1 and Th17 cells in the pathogenesis and advancement of autoimmune complications such as MS. Successful activation of CD4^+^ T cells requires the body to recognize major histocompatibility complex (MHC) class-linked antigens [62], including T cell antigens [62]. In MS and related disorders, T cell antigens are recognized in the central and peripheral parts of the nervous system. In both cases, researchers have cited the possible contribution of B cells and how they affect the functions of T cell effector molecules [63].

Researchers recently stated that the association between MS and B cells involves myelin-oriented antibody functioning [38]. The antibody category is determined via the actions of the autoreactive forms of B cells in the CNS. In addition, the generation process is enhanced through the effector T cells [38]. Evidence from multiple sclerosis pathology demonstrated the presence of T cells in the germinal centers and antigen-oriented cells in MS samples [63, 64]. The outcomes of these studies suggested that B cells affect MS via cortical neuronal damage and by influencing the production and functioning of myelin-oriented antibodies.

**Fig. 2:**
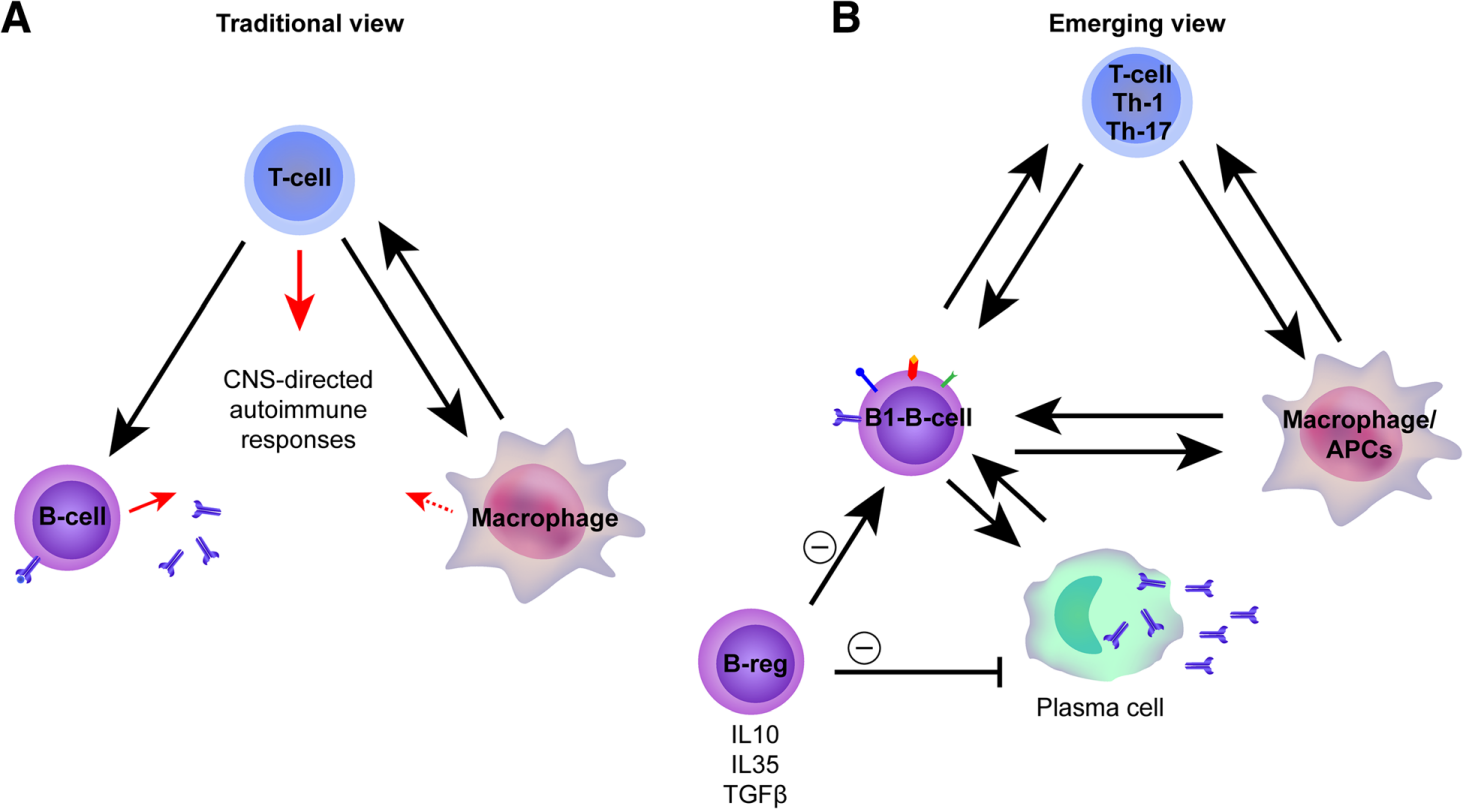
The evolving view of cell subset contributions to multiple sclerosis pathophysiology. **a** The traditional B cell view. T cells are central players in both MS immunological pathophysiology and the regulation of CNS-directed autoimmunity. An imbalance between proinflammatory, type-1 helper T cells (TH1), on the one hand, and TH17 effector T cells (Teff) and Treg cells, on the other hand, provokes new MS attacks. Myeloid cells, as the main antigen-presenting cells (APCs), shape the T cell responses. In turn, differentiated T cells can shape myeloid cell responses. B cells are a relatively homogenous and passive population. They await the help of T cells to differentiate into antibody-secreting plasmablasts and plasma cells. Any B cell contribution to MS pathophysiology is generally considered to reflect the potential of B cells to produce CNS-autoreactive antibodies. **b** The updated B cell view. In the updated view, the B cell is a full participant in a complex network. This network contains macrophages, T cells, and regulatory B cells. In MS, this complex network somehow became dysregulated. Furthermore, there are two well-established subcategories of B cells, the B1B cells and the B2B cells. For the autoimmune reactions that occur in MS patients, the B1B cells get out of control. Furthermore, the results of anti-CD20 (aCD20) therapy in MS suggest a more central role for B cells in new MS attacks, which often appear to be antibody independent. The antibody-independent action of B cells, partly mediated via the elaboration of distinct cytokines, can manifest as either proinflammatory effector B1B cells (B-1-B) or anti-inflammatory regulatory B cells (B-reg). These cells can activate the B-1-B or downregulate the B-reg proinflammatory responses of both T cells and myeloid cells. Bidirectional interactions among functionally distinct B cells, T cells, and myeloid cells—and the consequences of such interactions—provoke the development of new MS attacks

For the evolving new view on B cells in MS, see Fig. 2.

### Regulatory B cells and MS

Research suggests an interplay between T cell subsets, myelin-linked antibodies, and B cells that can affect the advancement and severity of MS [64]. In addition, this interplay affects the symptoms that patients will show at different phases of the disorder. The process appears to be controlled by the numerous B cell-based substances such as the B cell-activating factor (BAFF) molecule and CXCL13 [64]. Further evidence from studies examining the pathogenic purpose of subsets of B cells showed that their interaction with T cells can affect immune system functioning and plasma cell survival factors. In the end, this process affects the secondary advancement of MS [65]. The existence of ectopic follicles in the samples obtained from MS cases and populations suggests the possibility of B cell action and inflamed organ replication [64, 65]. In the later stages of the disorder, isolation of follicles from meningeal B cells further supports the role and connection between B cells and MS [65].

The three putative biological roles of B cells are antibody production, antigen presentation, and immunoregulatory cytokine production. The latter has led to the recognition of different B cell subtypes producing either proinflammatory or regulatory cytokines (B effector cells and regulatory B cells). These B cells can switch myeloid cells (and subsequently T cells) to a proinflammatory phenotype. After depletion with rituximab, repopulated B cells showed a reduced number of GM-CSF-producing B cells. The results from these studies suggest a possible connection between various B cells and MS development. The process appears to be aided by the production and careful regulation of T cells with a range of anti-inflammatory capabilities.

Studies have shown that the B cell groups can affect MS by exerting their regulatory properties [64, 65]. This process is modulated by IL-10 molecules [64, 65]. Experimental and research results have shown that non-activated B cell groups can regulate and control autoimmune responses in humans. In addition, recent investigations showed that IL-21-reliant processes may underlie the formation of B cells and IL-10 [66, 67]. Other studies have shown that B cells may aid in inhibiting TNF production in MS cases. Additionally, the presence of B cell biomarkers in MS patients suggests an ability to regulate proinflammatory activity in APCs [68]. These results show that B cell populations act as a significant source of plasma cells that generate antibodies while also regulating the autoimmune process through the production of anti-inflammatory T cells [69]. These cells influence the autoimmune system functions by regulating the release of molecules that can suppress APC activity [70]. These findings further show the complex nature of B cells and the diversity of their roles in the autoimmune system and MS.

### IgM i.v.–Ig therapy

MS is a severe demyelinating disorder that affects the CNS and adversely affects patients’ well-being. In some cases, patients experience a relapsing-remitting course due to the progression of their neurological disabilities. Therefore, interventions must be developed to manage the condition. Intravenous immunoglobulin (IVIG) is regarded as one therapy that can be used to manage MS. Previous studies indicated that this intervention can improve patients’ short-term and long-term well-being [40]. IVIG intervention consists of a mixture of antibodies that can improve immune function. In addition, these immunoglobulins can stimulate and suppress the immune system depending on the person’s disorder [45]. Presently, researchers lack conclusive evidence on the actual mechanism through which the therapy works [45]. However, the intervention is believed to dose-dependently downregulate B cell and T cell functions, a trend that improves the body’s response to immune-mediated disorders such as MS. Cross-linking different B cell antigens with IgM antibodies often leads to a cascade of processes that improve the immune response.

IVIG therapy may increase the chances of recovery from a relapsing-remitting MS course. Furthermore, the therapy can enhance the blood-brain barrier permeability, suppress gadolinium production and expression, and reduce demyelination rates among patients with MS [46, 47]. Although most patients can tolerate the therapy well, it may have adverse effects, including dizziness, nausea, and headaches. In some cases, the therapy may lead to infusion reactions and severe allergic reactions in patients and may promote the risk of severe adverse effects such as aseptic meningitis, arterial complications, and thrombosis. Despite this, IVIG remains a potential therapy that can assist in managing MS. In addition, IVIG may reverse the demyelination process, thereby enhancing the well-being and quality of life among MS patients.

IVIG’s immunomodulatory effects have been linked to different biological actions and functions of IgM in the body. In addition, the effects of the drugs are usually mediated by the antigen-binding F(ab′)_2_ and Fcμ parts of the IgM. Furthermore, IVIG has been linked to anti-inflammatory activities and the ability to bind to the inhibitory FcγRIIb receptor found in macrophages [50]. These complex processes may improve immune responses and contribute to slowing MS progression. In some cases, researchers have stated that the intervention works through IgM’s suppressive effect, clearing self-antigens, and inhibiting idiotypic interactions in the body [56]. However, further investigations are needed to explore the exact mechanism through which the therapy improves the well-being of patients with MS.

## Discussion

MS is a severe autoimmune disorder that can affect an individual’s health and well-being. Coping with the disorder is a transactional process that depends on many factors and changes over time [38]. The process can be affected by personal, environmental, and temporal issues that influence MS development. For healthcare practitioners to help patients recover and regain lost function, they must accurately identify and understand the factors that cause MS [45]. In addition, comprehending the patients’ concerns gives healthcare practitioners and researchers critical insights that can improve care decisions.

B cells have been identified to be among the likely factors that affect MS development and advancement. The actual mechanism through which the cells facilitate MS emergence remains unclear. However, previous studies have suggested that the cells may aid MS emergence by regulating the autoimmune system, acting as a source of antibody-producing plasma cells, and functioning as APC controllers [68, 69]. In other cases, researchers examined the cells’ roles by focusing on how they foster the production of pro- and anti-inflammatory molecules and cells and influence APC activity. While studies have provided important insights into the purposes of B cells, they also paint a complex picture of the link between B cells and MS [70]. Despite this, B cells present important targets that can guide the development and use of therapies to manage MS.

## Conclusion

The true cause of MS remains unknown. However, research has implicated several biological, genetic, and environmental factors. The current review examined the role of B cells in MS development and progression. Evidence from previous studies suggests a complex relationship between B cell groups and MS. Furthermore, research indicates that B cells aid MS pathogenesis by influencing and regulating different autoimmune processes such as T cell production and APC activity. Studies have revealed that recruitment and activation of autoimmune B cells are central to MS development and progression. B cells produce distinct molecules that influence the manner in which the innate immune system reacts to the disease. Further studies are needed to examine the definite mechanism that underlies the relationship between specific B cell categories and MS.

## Data Availability

All data are given in the manuscript.
